# Chronic demyelination interferes with normal spermatogenesis in cuprizone-intoxicant C57/BL 6 mice: An experimental study

**DOI:** 10.18502/ijrm.v22i1.15241

**Published:** 2024-02-23

**Authors:** Arezoo Dorikhani, Ameneh Omidi, Mansoureh Movahedin, Iman Halvaei

**Affiliations:** Department of Anatomical Sciences, Faculty of Medical Sciences, Tarbiat Modares University, Tehran, Iran.

**Keywords:** Multiple sclerosis, Cuprizone, Hypothalamic-pituitary-gonadal axis, Spermatogenesis.

## Abstract

**Background:**

Due to myelin and axonal insults in multiple sclerosis individuals, motor coordination problems and endocrine imbalance may develop.

**Objective:**

This study aims to evaluate the role of chronic demyelination on the hypothalamic-pituitary-gonadal axis in the mouse model of multiple sclerosis.

**Materials and Methods:**

20 adult C57/BL6 male mice were divided into 2 groups (n = 10/each) as follows: the control group (CONT) received a regular diet for 17 wk; and the experimental group (cuprizone [CPZ]) was fed with 0.2% CPZ for 12 wk and, then CPZ was withdrawn for 5 wk. Serum testosterone, histopathology of the brain and testis, and sperm analysis were evaluated.

**Results:**

The hypothalamic myelin content was significantly decreased in the arcuate nucleus following the 12 wk of CPZ consumption compared to the CONT group, and the statistical difference remained until 17 wk. Testosterone levels declined significantly in the CPZ group compared to the CONT group in the 12 ${\;}^{\text{th}}$ and 17 ${\;}^{\text{th}}$ wk. A significant decrease was observed in the height of the seminiferous epithelium and the interstitial tissue area, and the number of seminiferous epithelial cells in the CPZ group compared to the CONT group in the 12 ${\;}^{\text{th}}$ and 17 ${\;}^{\text{th}}$ wk. The sperm count, motility, and viability in the CPZ group significantly decreased compared to the CONT group in the 12 ${\;}^{\text{th}}$ and 17 ${\;}^{\text{th}}$ wk of the study.

**Conclusion:**

Chronic demyelination induced by CPZ intoxication, maybe through damage to the hypothalamus arcuate nucleus, leads to the hypothalamic-pituitary-gonadal axis disturbance and damage to the testis and spermatogenesis subsequently.

## 1. Introduction

Multiple sclerosis (MS) is a chronic demyelinating neurological disorder characterized by selective myelin degradation and axonal damage resulting in reduced synaptic diffusion (1). Numerous factors, such as immune-related disorders, infectious diseases, and illnesses related to toxic and metabolic substances, can lead to demyelination diseases (2). Depending on the lesion site, MS individuals experience various clinical features like sensory challenges, motor coordination disturbances, and endocrine defects (3). Regulating the reproductive system is mainly owed to the normal function of the hypothalamic-pituitary-gonadal (HPG) axis (4). Therefore, any factor that disrupts the axis could affect the production of gonadal hormones and reproductive function (5). However, the exact pathophysiology of endocrine disturbances in demyelination diseases and the involvement of the HPG axis is still poorly understood (4). The testosterone level is regulated by endocrine interactions between the hypothalamus, anterior pituitary gland, and testis (6). The responsible neurons for gonadotropin-releasing hormone (GnRH) secretion frequently concentrate in the arcuate nucleus of the hypothalamus (7).

Although hormonal and sexual disorders are common symptoms in many neurological diseases (8), due to several factors like the complex nature of these symptoms and the influence of other symptoms, sexual and fertility issues are an MS individual challenge that needs more attention and investigation (9). Several hormonal disturbances are the main culprits of men's fertility rate impairment. Epidemiological data shows that sexual dysfunction, such as orgasmic, erectile, and ejaculatory disturbance, is manifested in an approximately large population of MS men (10).

Since testosterone metabolism is disrupted in men with MS, the formation of lesions in the hypothalamus may affect sperm production, semen quality, and sexual function by disrupting the HPG axis (4). Serum testosterone levels significantly diminished in experimental autoimmune encephalomyelitis male mice in a way that there was an inverse relationship between the inflammatory cytokines and hormone levels (11). A study on the HPG axis and sperm parameter analysis reported that mean testosterone level, sperm count and motility, and normal sperm morphology in men with MS were significantly decreased (12).

Cuprizone (CPZ) is one of the most widely used neurotoxicants to induce the MS model in mice (13). By disrupting the copper-dependent cytochrome oxidase activity and reducing oxidative phosphorylation, CPZ leads to oligodendrocyte apoptosis (14).

The induction of the demyelinated animal model may be an appropriate and necessary demand for evaluating more comprehensive studies related to reproductive system issues in MS individuals. Due to the lack of previous studies on histopathology of the hypothalamic region and also less attention paid to the fertility issue in men with MS, this study investigated the impacts of hypothalamus demyelination on the testosterone, spermatogenesis, and sperm parameters as the manifestations of the HPG axis, in the CPZ animal model of MS.

## 2. Materials and Methods

### Animals and grouping 

In this experimental study, 20 adult C57/BL6 male mice (5-wk-old, 18–20 gr) were prepared from Pasteur Institute (Karaj, Iran). After 1 wk of adaptation to the environment, the animals were divided into 2 groups randomly (n = 10/each). The first group received a regular diet for 17 wk and was considered as control group (CONT). The second group received a diet containing 0.2% CPZ (Sigma-Aldrich, USA) for 12 wk to induce chronic demyelination, and then CPZ was removed from their diet for 5 wk to give the animal sufficient time for spermatogenesis (CPZ) (15).

### Measuring serum testosterone

To measure the testosterone level of serum, the control and CPZ animals were deeply anesthetized at day zero and at the end of the 12 ${\;}^{\text{th}}$ and 17 ${\;}^{\text{th}}$ wk by intraperitoneal injection of 100 mg/kg body weight ketamine (Sigma-Aldrich, USA) and 10 mg/kg body weight xylazine (Sigma-Aldrich, USA). The blood sample was drawn directly from the heart, and then followed by the centrifuge (at 3000 rpm for 10 min), the resulting serum was collected. Finally, serum testosterone level was measured by electrochemical luminescence technique using an available commercial kit (Roche, Germany) (12).

### Histopathological studies of the brain

The brain sample was placed in a 4% paraformaldehyde fixative solution (Merk, Germany). Following dehydration in tissues with alcohols of different purity, the tissues were clarified with xylol and finally blocked with paraffin. 5-µm thick sections stained with luxol fast blue-periodic acid Schiff staining (Alfa Aesar, USA) to evaluate the hypothalamus demyelination. The desired brain sections of the hypothalamic arcuate nucleus were acquired using the Paxinos and Franklin's mouse brain atlas in bregma -3.12 mm (16). Demyelination intensity and myelin density in 20 regions with dimensions of 250 × 250 µm were evaluated using Digimizer software (version 5.4.7, MedCalc Software Ltd., Ostend, Belgium) (15).

### Weight examination and histopathological studies of testis 

Following removing the surrounding tissue, testis weight was measured, and the water immersion method was applied for the volume gauged. It should be noted that in addition to the 12 ${\;}^{\text{th}}$ and 17 ${\;}^{\text{th}}$ wk, the mouse testes were weighted on day zero. Following infiltration of Bouin's solution (Sigma, USA) as a fixative, each testis was cut longitudinally and divided into 2 parts. After dehydration using alcohol, the tissue was clarified by xylol and finally blocked with paraffin. Then, the 5 µm-thick sections were prepared by rotary microtome device (Dide Sabz, Iran) and placed on slides impregnated with albumin glue. Finally, hematoxylin and eosin staining (Sigma Chemical Co., USA) were used for morphometric evaluation of testis tissue (17).

### Testicular tissue morphometry

For morphometrical evaluations of the testis, obtained images (x40 magnification) were assessed using ImageJ software (version 1.8.0, National Institutes of Health, USA). We examined 100 fields in 5 defined-intervals sections prepared per testis. For the diameter of the seminiferous tubule, the large and small round or relatively round pipe diameters were measured, and then the average of these 2 diameters were recorded. With the subtraction of the outer and inner diameters of seminiferous tubules, the epithelial thickness of the tubules was obtained. In addition to counting desired epithelial cells, the total testicular interstitial area was acquired by subtracting all seminiferous tubule areas from the field area (17).

### Evaluating sperm parameters 

At the end of the 12 ${\;}^{\text{th}}$ and 17 ${\;}^{\text{th}}$ wk of the study, following complete anesthesia, a longitudinal incision was made in the left scrotum of the mice. After removing the skin and fascia, the testicle appeared, and by a small incision in the surrounding sheath, the epididymis appeared thoroughly to be used to analyze sperm parameters (17).

#### Sperm sampling and analysis

To achieve the animal sperm, the left caudal region of the epididymis was isolated and placed in a 3 cm plate containing 1 mL of culture medium (Dulbecco's Modified Eagle Medium) (Gibco, USA) containing 10% fetal bovine serum (Gibco, USA). Before surgery and to provide suitable conditions for sperm, the culture medium was incubated overnight at 37 C with 5% CO ${\;}_{2}$ . After placing the tail of the epididymis in the plate, small incisions were made in the tissue sample using an insulin syringe to facilitate sperm release. Then, the sperm was incubated for 1 hr to stabilize and prepare them for analysis. Noteworthy, to homogenize the medium and uniform distribution of the sperms throughout it, gentle pipetting was performed. For sperm analysis, the desired items, including viability, morphology, sperm count, and motility, were performed according to the World Health Organization (17).

#### Sperm count

For counting the sperms, based on the standard Neubauer slide method, after diluting a medium containing spermatozoon with 2% formaldehyde fixative, 10 µl of the sample was transferred on a Neubauer slide with a placed coverslip on it. Sperms with a head, middle piece, and tail were counted under a light microscope (Labomed, USA) (x40 magnification). The counting of sperm was calculated based on World Health Organization guidelines regarding sperm concentration at a cubic milliliter (17).

#### Sperm motility

The total motility of the sperm and its speed of movement was determined. At first, 10 µl of culture medium containing sperm was transferred to a slide, and at least 200 sperms were examined under a light microscope at x40 magnification. Motility was observed and recorded as motile or immotile sperms, and in turn, the motile sperms, based on their movement style, were categorized as progressive and non-progressive. For each sample, after repeating twice, the average was recorded and finally reported in percentage (17).

#### Sperm viability 

The viability of sperm was assessed by eosin-nigrosin staining (Merk, Germany). Based on this staining, the head of dead sperms turns red or dark pink, but the head of live sperms remains colorless. At first, the 1:2 ratio (by volume) of sperm suspension and eosin was mixed in the microtube. Following 30 sec, an equal volume of nigrosin solution was added. Then, a thin spread of the sample took place on the slide. After drying the smear, using a light microscope (100x magnification), 200 sperms per sample were counted. Eventually, the percentage of live and dead sperms in the samples was calculated (17).

#### Sperm morphology

Papanicolaou staining (Merk, Germany) was used to evaluate sperm morphology. The smear was prepared from each sample and placed at room temperature for 3 hr to dry completely. Then, it was immersed in 95% alcohol for 20–25 min and dried again to prepare for staining. Then, slides were dried in the presence of air, and after drying, 400 sperm were counted in each sample under a light microscope at x100 magnification (17).

### Ethical considerations

Approval was received from the Ethics Committee of Tarbiat Modares University, Tehran, Iran (Code: IR.MODARES.REC.1400.207). All the procedures were carried out under the supervision of the committee and in accordance with the animal laboratory principles.

### Statistical analysis 

The Kolmogorov-Smirnov test was used to assess the data normality. Repeated measure ANOVA and post hoc LSD tests were used for intragroup comparison of testosterone levels. 2-independent sample *t* test and Mann-Whitney U test were used for intergroup comparison (for normal and abnormal data distribution, respectively). The significance differences level was considered p < 0.05. All statistical operations were performed using SPSS software (version 25.0, SPSS Inc., USA), and graphs were drawn by GraphPad Prism software (version 8.0.2, GraphPad Software Inc., San Diego, CA, USA).

## 3. Results

### The effect of chronic demyelination on serum testosterone level

**Figure 1:**
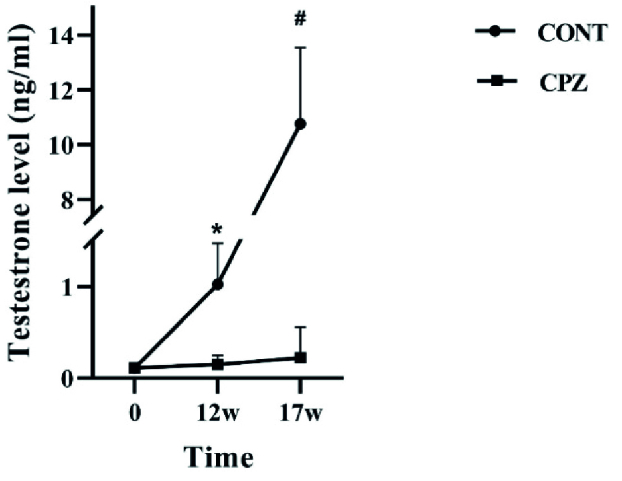
Testosterone measurement (ng/ml). CONT and CPZ groups. CONT: Control, CPZ: Cuprizone, *and ${\;}^{\#}$ shows the p < 0.05.

The results of measuring serum testosterone levels showed that in the CONT group, hormone levels increased significantly in the 17 ${\;}^{\text{th}}$ wk compared to day zero and the 12 ${\;}^{\text{th}}$ wk (p = 0.02). Surprisingly, no significant differences were observed between the 3-time points in the CPZ group. The data revealed that the level of testosterone in the CPZ group was significantly lower than in the CONT group in the 12 ${\;}^{\text{th}}$ and 17 ${\;}^{\text{th}}$ wk (p = 0.03, and p = 0.02, respectively) (Figure 1).

### Histopathological evaluations of the brain

**Figure 2:**
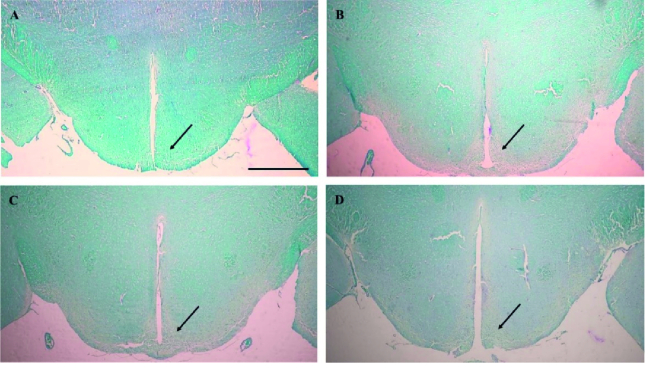
Luxol fast blue-periodic acid Schiff staining (PAS/LFB) staining in the hypothalamic arcuate nucleus (bregma: -3.12 mm). A) CONT group in the 12 ${\;}^{\text{th}}$ wk, B) CPZ group in the 12 ${\;}^{\text{th}}$ wk, C) CONT group in the 17 ${\;}^{\text{th}}$ wk, and D) CPZ group in the 17 ${\;}^{\text{th}}$ wk. Black arrows indicate the arcuate nucleus of the hypothalamus. The fully myelinated regions are observed as dark blue staining, like what is obviously seen in part A, while the demyelination areas are displayed as pale blue staining, like what is remarkably observed in part B. Scale bar = 300 µm.

**Figure 3:**
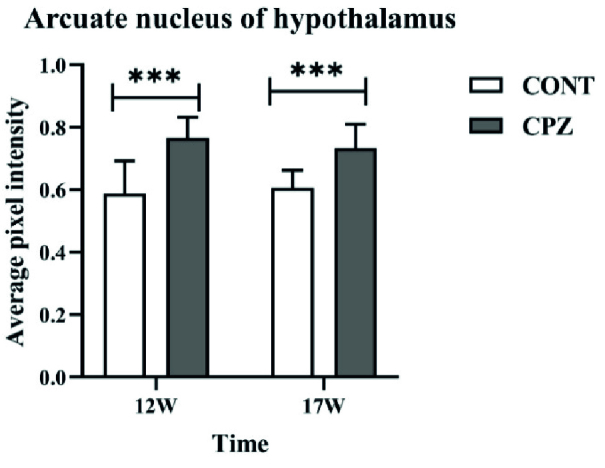
The demyelination of the hypothalamic arcuate nucleus. Bregma: -3.12 mm. ***(p < 0.001). CONT: Control, CPZ: Cuprizone.

Histopathological results by digimizer software showed that the demyelination of the hypothalamus arcuate nucleus in the CPZ group was significantly higher compared to the CONT group in the 12 ${\;}^{\text{th}}$ and 17 ${\;}^{\text{th}}$ wk (p < 0.001) (Figures 2 and 3).

### Testicular weight examination

**Figure 4:**
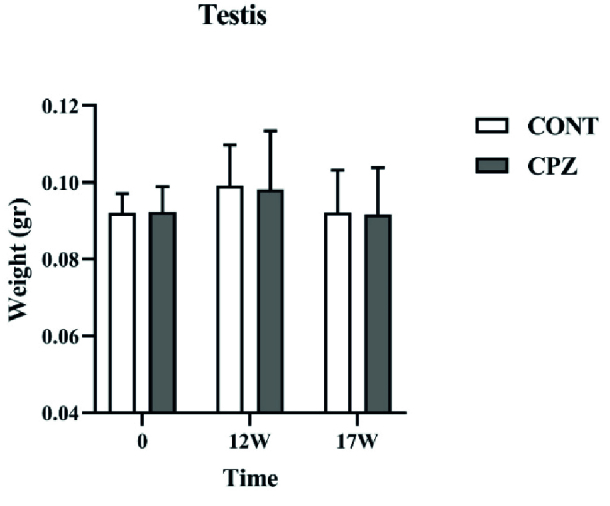
Testis weight (gr). CONT: Control, CPZ: Cuprizone.

Measuring the testis weight showed no significant difference between the CONT and CPZ groups on day 0, week 12, and week 17 of the study (Figure 4).

### Histopathological and morphometric data of testis

**Figure 5:**
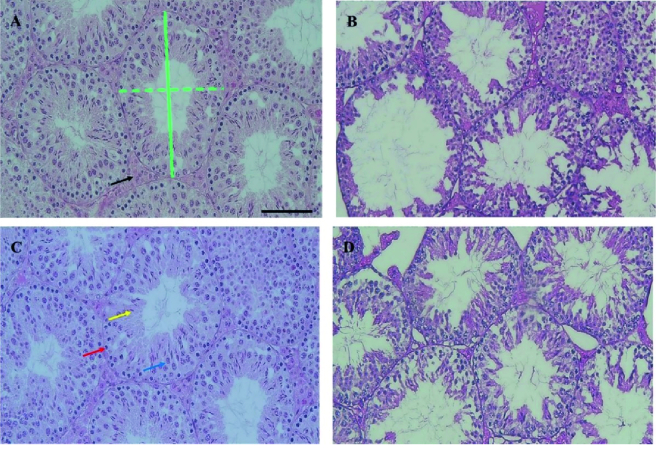
Hematoxylin and eosin (H&E) staining of the testis. A) CONT group in the 12 ${\;}^{\text{th}}$ wk, B) CPZ group in the 12 ${\;}^{\text{th}}$ wk, C) CONT group in the 17 ${\;}^{\text{th}}$ wk, D) CPZ group in the 17 ${\;}^{\text{th}}$ wk. Green lines: Diameter of the seminiferous tubule (the average of the large and small diameters [solid and dashed green lines, respectively]), black arrow: Interstitial tissue area, red arrow: Spermatogonia, blue arrow: Spermatocytes, yellow arrow: Spermatid, CONT: Control, CPZ: Cuprizone. Scale bar = 50 µm.

**Figure 6:**
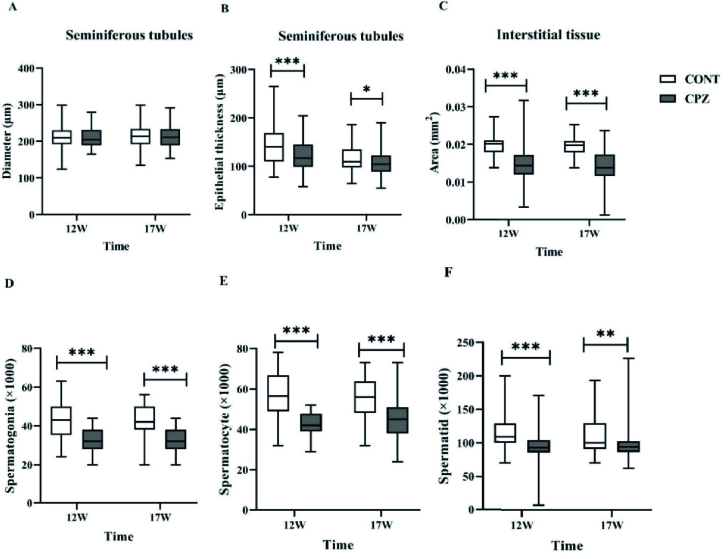
Histomorphometric evaluations of the testis. A) The diameter of the seminiferous tube (µm), B) The thickness of the epithelium of the seminiferous tube (µm), and C) The area of interstitial tissue (mm^2^), D) The number of spermatogonia cells, E) The number of spermatocytes, F) The number of spermatid cells. *, ** and ***displays the significant difference (p < 0.05,p < 0.01, and p < 0.001, respectively). CONT: Control, CPZ: Cuprizone.

Complete and regular seminiferous epithelium were observed in the seminiferous tubules of the CONT group in the 12 ${\;}^{\text{th}}$ and 17 ${\;}^{\text{th}}$ wk. Although in the CPZ group, disruption of seminiferous tubules occurred, the diameter of the seminiferous tubules showed no significant differences between groups. The results showed that the epithelial thickness of the seminiferous tube in the CPZ group was significantly lesser compared to the CONT group in the 12 ${\;}^{\text{th}}$ (p < 0.001) and 17 ${\;}^{\text{th}}$ (p = 0.02) wk. The data showed that the interstitial tissue area in the CPZ group was significantly lower than the CONT group in the 12 ${\;}^{\text{th}}$ and 17 ${\;}^{\text{th}}$ wk (p < 0.001). The number of both spermatogonia and spermatocytes in the CPZ group was significantly lower compared to the CONT in the 12 ${\;}^{\text{th}}$ (p < 0.001) and 17 ${\;}^{\text{th}}$ wk (p < 0.001). In addition, the number of spermatid cells in the CPZ group was significantly lower compared to the CONT in the 12 ${\;}^{\text{th}}$ and the 17 ${\;}^{\text{th}}$ (p < 0.001 and p = 0.008, respectively) (Figures 5 and 6).

### Sperm analysis 

**Figure 7:**
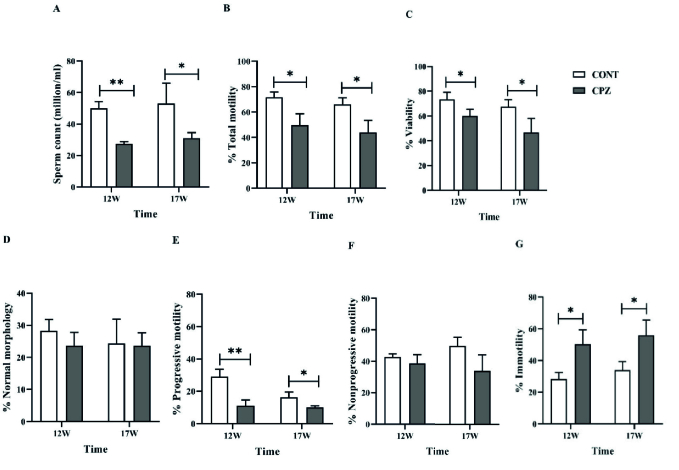
Sperm analysis. A) The number of sperms in the semen (million/ml), B) Percentage of total motility, C) Assessment of sperm viability,D) Percentage of normal morphology of sperms, E) Percentage of progressive motility of sperms, F) Percentage of non-progressive sperm related to motility test, G) Percentage of immotile sperms. *and **indicate the statistically significant difference (p < 0.05 and p < 0.01, respectively). CONT: Control, CPZ: Cuprizone.

The concentration of sperms in the CPZ group decreased significantly compared to the CONT group in the 12 ${\;}^{\text{th}}$ and 17 ${\;}^{\text{th}}$ wk (p = 0.001 and p = 0.04 in order). In addition, the sperm total motility in the CPZ group decreased significantly compared to the CONT group in the 12 ${\;}^{\text{th}}$ and 17 ${\;}^{\text{th}}$ wk (p = 0.01 and p = 0.02, in order). Although the rate of progressive sperm in the CPZ group decreased significantly compared to the CONT group in the 12 ${\;}^{\text{th}}$ and 17 ${\;}^{\text{th}}$ wk (p = 0.006 and p = 0.03, respectively), no statistical differences were observed in non-progressive percentages between groups. Immotile sperm percentage in the CPZ group increased significantly compared to the CONT group in the 12 ${\;}^{\text{th}}$ and 17 ${\;}^{\text{th}}$ wk (p = 0.01 and p = 0.02, respectively). The percentage of non-motile sperms and motile sperms (total motility) was recorded. In addition, sperm viability significantly declined in the CPZ group compared to the CONT group at the 12 ${\;}^{\text{th}}$ and 17 ${\;}^{\text{th}}$ wk (p = 0.04). No significant difference was observed in the rate of sperm normal morphology between the 2 groups (Figure 7).

## 4. Discussion

This study investigates the deteriorative effects of chronic demyelination on the HPG axis in the male C57/BL 6 mice. Testosterone, the pivotal and essential regulator of the male normal sexual and reproductive function, significantly declined in the CPZ-intoxicant animals. In addition to the hypothalamic demyelination, the testis' regular histology, as well as sperm parameters, were dramatically affected in the animal model of MS.

Problems related to endocrine and sexual dysfunction that consequently interfere with spontaneous fertility are one of the highly prevalent symptoms in MS individuals that intensively impact the quality of life (5, 18). Secondary to the hypothalamic lesions, like what happens in MS disease, disturbances of the HPG axis occur that lead to hormonal dysregulation and may contribute to fertility problems. However, the effects of low testosterone levels on spermatogenesis in MS individuals are unclear, and further investigations are required to elucidate the neuro-sexual issues (19).

Since the development of spermatogonia to the point of spermiation in mice is more rapid than in humans, in a way that the effective generation of sperm needs 35 days (20). In our study following induction of chronic demyelination (12 wk), the essential time (5 wk) was considered to resume spermatogenesis in C57/BL 6 mice. The serum testosterone levels decreased dramatically following 12 wk of CPZ intoxication and remained low even until 5 wk of CPZ withdrawal. Although several studies show a dramatic decrease in testosterone levels in males with MS (11, 12). A study exploring the relationship between sexual hormones and the extent of brain insults in 26 MS men revealed no statistically significant decrease in serum testosterone levels. However, a positive correlation was observed between estradiol concentration and brain damage (21). This controversy in testosterone levels in different MS-related studies may be attributed to features such as location, number, and the activity mode of the MS lesions. However, the significant decrease in testosterone levels following chronic demyelination, even 35 days after CPZ removal, may be attributed to the hypothalamus and HPG axis deteriorations in the C57/BL 6 mice.

Contrary to the well-known and fundamental role of the hypothalamus, a very small number of studies have investigated the functional role of demyelination of hypothalamus nuclei in MS individuals (22). To further elucidate the roles of HPG axis disturbances, the arcuate nucleus of the hypothalamus was histologically examined in the chronic demyelination mouse model. The evidence showed that severe demyelination in the arcuate nucleus occurred in the following 12 wk of CPZ intoxication, and the demyelination remained until 17 wk. The neurons of the arcuate nucleus by some key regulators, such as kisspeptin and neurokinin B in the hypothalamus, are involved in the release of GnRH decapeptide and its transport to the pituitary portal circulation (23, 24).

Neuroimaging analysis showed the demyelinating lesions in the hypothalamus and revealed a significant reduction in the total volume of the hypothalamus and its subunits in MS individuals (22). In addition, brain MRIs disclose that individuals with active MS have a higher frequency of lesions in their hypothalamus (25). Probably, demyelination of the arcuate nucleus, which is a fundamental nucleus in GnRH secretion, has a pivotal impact on the lowering testosterone level in the C57/BL 6 mice.

Although there have been studies on the devastating effects of MS disease on testosterone secretion, limited studies on the MS impacts on testicular tissue seem to be available. In this study, the deteriorative effects of chronic demyelination with CPZ on testicular tissue have been discussed for the first time. In a study following bisphenol A exposure in the encephalomyelitis animal model, a small amount of apoptosis was observed in the individual spermatogonia cells, and no statistically significant changes in testis weight were seen (26).

In agreement with our results, testis weight did not significantly change after CPZ intoxication. In this study, following 12 wk of CPZ exposure, the testis of C57/BL 6 mice underwent destructive tissue damage so that the epithelium thickness and the area of the interstitial tissue, and also the number of testicular epithelia including spermatogonia, spermatocyte, as well as spermatozoa were significantly decreased. These testicular tissue insults remained significant until the 17 wk, which may be attributed to the demyelination effects of the hypothalamus and HPG axis disturbances and lack of enough testosterone secretion to maintain the testicular epithelium and interstitial tissue. However, testicular tissue damage in MS individuals or even in the animal models of MS has not been adequately addressed in the previous studies, so more research is needed.

To respond to the question of whether chronic demyelination impacts sperm parameters such as viability and motility, the CPZ intoxication as a well-acceptable animal model that relatively mimics the demyelination aspects of MS in the absence of other confounding immune responses (15) was applied. However, the effects of disease-modifying therapies in MS on semen have been investigated rather than exploring the destructive neuropathological impacts of MS on sperm parameters (5). Anyway, despite the relatively easy availability of semen, there is limited data on male infertility's association with MS (27). Sperm parameters such as sperm count, motility, and the percentage of normal-shaped sperms have declined in MS individuals, which was attributed to the disturbance in the hypothalamic-pituitary-testis axis (12). In addition, transient or even permanent azoospermia was found in a significant percentage of MS individuals receiving disease-modifying therapies (5).

The results of the current study revealed that the sperm count and viability percentage, as well as total and progressive motility percentages, were significantly decreased following chronic demyelination and even 5 wk of CPZ withdrawal in the C57/BL 6 mouse model.

However, despite sexual dysfunctions being one of the common complaints of MS individuals (28), because of various items like considering sexual deficits as a secondary symptom (3), these issues are highly underdiagnosed (29) and mostly under-investigated (9). Concerning the deteriorative consequences of demyelination on endocrine/sexual and fertility issues, more experimental and clinical investigations should consider these complications in MS individuals (30).

## 5. Conclusion

The finding of the current study reveals that beyond the endocrine disturbance, the chronic demyelination led to disruptions in sperm parameters as well as the histopathological insults of the testis in the CPZ-intoxicant C57/BL 6 mice. The persistent deteriorative effects of chronic demyelination, even 5 wk of CPZ withdrawal, could be more intensively attributed to the HPG axis disturbances via the arcuate nucleus of hypothalamus demyelination.

##  Data availability

Data supporting the findings of this study are available upon reasonable request from the corresponding author.

##  Author contributions

Arezoo Dorikhani: investigation, data curation, writing the original draft of the article; Ameneh Omidi: conceptualization, supervision, funding acquisition, data analysis, writing, reviewing, and editing the article; Mansoureh Movahedin: conceptualization, supervision; Iman Halvaei: methodology. All authors approved the final manuscript and take responsibility for the integrity of the data.

##  Conflict of Interest

The authors declare that there is no conflict of interest.
